# The Effects of Hydrocolloids-Protein Mixture as a Fat Replacer on Physicochemical Characteristics of Sugar-Free Muffin Cake: Modeling and Optimization

**DOI:** 10.3390/foods10071549

**Published:** 2021-07-05

**Authors:** Elahe Azmoon, Farzad Saberi, Fatemeh Kouhsari, Mehdi Akbari, Marek Kieliszek, Amir Vakilinezam

**Affiliations:** 1 Department of Food Science and Technology, Science and Research Branch, Islamic Azad University, Tehran 14778-93855, Iran; azmoon.elahe@gmail.com; 2 Department of Research and Development, Zarkam Company, Zar Industrial and Research Group, Hashtgerd 33611-18549, Iran; f.kouhsari@zarholding.com (F.K.); a.vakili@zarholding.com (A.V.); 3 Department of Food Science, Engineering and Technology, College of Agriculture and Natural Resources, University of Tehran, Karaj 31587-77871, Iran; 4 Department of Food Biotechnology and Microbiology, Institute of Food Sciences, Warsaw University of Life Sciences—SGGW, Nowoursynowska 159 C, 02-776 Warsaw, Poland

**Keywords:** sugar-free and low-fat cake, konjac gum, guar gum, soy protein isolate

## Abstract

The purpose of this study was to evaluate the hydrocolloids–protein mixture as a fat replacer in sugar-free low-fat muffin cakes. In this study, a hydrocolloids mixture including konjac and guar gums and soy protein isolate (SPI) was applied to the cake. The combination of gums and SPI was named as mixture of stabilizers (MOS), and the treatments were designed using Design-Expert software and the response surface methodology (RSM) in order to optimize and reduce the oil content of muffin cakes by replacing it with MOS. Evaluation of treatments characteristics were investigated on days 1 and 15 of their production. The dependent variables were moisture content, water activity, specific volume, porosity, hardness, cohesiveness, springiness, chewiness and crumb color of cakes. The results show that increasing the percentage of MOS has positive effects on the final products in comparison to oil. In other words, an increase in the MOS content resulted in an increase in the moisture content, water activity, specific volume, height, springiness, cohesiveness, chewiness and L* (lightness) values, but negative effect on hardness, a* (redness) and b* (yellowness) values. As a result of optimizing using RSM, the usage of 4.08% oil and 0.31% MOS resulted in a 62.9% reduction in oil content in comparison with the control sample. The panelists assigned the lowest score to hardness and crumb color and the highest score to overall acceptability and cohesiveness to the optimized muffin.

## 1. Introduction

Nowadays, the application of fat replacers such as proteins and hydrocolloids has obtained much attention in the confectionary and bakery industries due to their effects on human health [1]. Overall, fats and oils have important functions in bakery products such as volumizing, tenderizing, machinability, performing softness and moistness, as well as enhancing mouth feel and taste [2]. However, many studies show that the high consumption of fats and oils leads to health issues among consumers. As mentioned before, amongst different materials, hydrocolloids and proteins are the most important ingredients in bakery products as fat replacers because of their high water binding and holding capacity. In low-fat cake and muffin formulations, hydrocolloids could be used widely to provide body, mouth feel, stabilize the emulsions and inhibit the syneresis phenomenon. Proteins and carbohydrates may provide their fat-replacing functionalities through two approaches. Firstly, carbohydrates and proteins are both macromolecules that yield physicochemical and sensory properties such as viscosity and thickening property, which may provide a fat-depleted product with properties comparable to products containing fats. Secondly, they can naturally form or be processed to form micro-particles with shapes and sizes similar to those of fat globules and emulsion droplets. Hence, they can be used to mimic fats [3]. Combination of different hydrocolloids can be applied for achieving the best synergistic properties. A number of studies have been presented on the usage of guar gum in low-fat bakery products focusing on the changes in rheological properties, improving water binding capacity, highly compatible synergistic attributes, storage stability and extending of shelf life [3]. These studies mostly focused on xanthan, locust bean and konjac gums or a combination of them, where xanthan gum was the most frequent gum used [4,5,6]. It should be mentioned that guar gum has not been used directly as a fat replacer in low-fat food recipes. In fact, it has been used as the agent to control the viscosity of batter, texture and water binding capacity [7]. Konjac gum, or konjac glucomannan, is another hydrocolloid which has been applied as a fat replacer. It is a polysaccharide that consists of D-gluctose and D-mannose with p-1, 4 glycosidic linkages obtained from the roots of *Lasioideae Amorphophallus* plant. Konjac gum has prominent features such as simulating fat characteristics in low-fat recipes, especially in low-calorie meat products [8]. Konjac gum can also be used in low-fat food formulations to make a gel structure with other ingredients, such as carrageenan and starch, as fat replacers [9]. In other studies, protein–carbohydrate complexes have been investigated in food formulations as fat replacers over the past years. The usage of protein–carbohydrate complexes in low-fat formulations has many benefits in their organoleptic properties, such as improving texture and mouth feel desirability [10,11]. Soy protein isolate (SPI) is commonly used in food systems and formulations to increase baking efficiency, reduce weight loss, improve the viscosity and emulsion stability. Adding SPI to bakery products can be accompanied by improved uniformity, softening and also stickiness reduction in the batter. It was found that final bakery products containing SPI will have better color, texture and symmetry [12]. Generally, SPI is preferred for use rather than other types of proteins, since it contains more than 90% protein in comparison to gluten, which contains 70% protein, and also shows no shrinking properties, similarly to gluten.

Muffins belong to the group of pound cakes which can contain up to 18–40% fat based on flour [13,14]. The addition of chia seed mucilage in bread [15], the usage of cellulose ether emulsion [16], inulin [17], xanthan and guar gums, soluble and insoluble fiber [18], succinyl chitosan [19], the addition of berry pomace as a fat replacer in cake [20], modified cassava starch [21] and extruded wheat flour [22] have been studied in the research which has been published on this topic. It is known that hydrocolloids in combination with each other show a synergistic effect. It is believed that konjac gum in combination with SPI will result in improved texture quality. Although much research has been done on the application of hydrocolloids and proteins as fat replacers, to the best of our knowledge, none has been performed that relates to the modeling and optimization of physicochemical properties of cakes using the recent mixture of hydrocolloids and protein [23,24,25].

Sucrose is the main sweetener in cakes and muffins. Sucrose acts as a tenderizer and stabilizer by increasing the egg protein denaturation and starch gelatinization temperature, and can contribute to cake volume [26]. However, the substitution of sugar in cake products has become a preference owing to consumer demands for dietetic alternatives. Polyols act as sugar substitutes because they are low in calories and have a low glycemic index [27]. Ronda et al. [28] reported that xylitol was the best substitute for sucrose, due to its similar hygroscopicity and sweetness level, while its sensory scores were close to the control sample.

Therefore, the aim of this study is to evaluate the combination of guar and konjac gums and SPI to create a novel and sustained emulsion which could replace the oil in the recipe of muffins by 60–80% to reduce the fat content of the formulation and achieve similar rheological and organoleptic characteristics to those of fully oiled muffin by means of the optimization of the ratios of the stabilizer mixture. For instance, this research may be considered as a novel formulation for a reduced-fat cake.

## 2. Materials and Methods

### 2.1. Materials

For cake preparation, wheat flour (13.5% moisture content, 11.4% protein (N × 5.7), 0.5% ash) and gluten were purchased from Setareh Co. (Karaj, Iran). The remainder of ingredients—xylitol and liquid sorbitol (Roquette Paris, France), sunflower oil (Oila Co., Eshtehard, Iran), whole pasteurized egg (Telavang, Tehran, Iran), glycerin (Cargill, Frankfurt, Germany), cake gel (Gilk 60, Aromatic, Sweden), baking powder, guar and konjac gums and isolated soy protein and potassium sorbate (Sigma Aldrich Co., St. Louis, Missouri, USA), flavoring agents (Firmenich Co., Geneva, Switzerland), and salt (Taban Co., Tehran, Iran) were purchased from their suppliers.

### 2.2. Emulsion Preparation

To prepare different emulsions, the mixture of isolated soy protein, konjac and guar gums at a fixed ratio of 10:4:3 wt. was treated according to pre-treatments which were performed mostly based on the research conducted by Vélez Erazo [29]. During the pre-treatments, the ratio of isolated soy protein was considered as fixed and the percentages of konjac gum and guar gum were considered as variables. Therefore, the ratio of konjac gum to guar gum was considered as 4:3, 3:4, 4:1, 1:4, 1:2 and 2:1, among which, the ratio of 4:3 was ultimately seen to be the best. The mixture was dispersed in sunflower oil and stirred using thermomix-vorwerk (TM5, Wuppertal, Germany) at 3000 rpm for 1 min. Then, water was added to the previously prepared mixture and completely blended for another 2 min in the abovementioned mixing conditions until a homogenous and sustained emulsion was obtained.

### 2.3. Batter and Cake Preparation

The control cake sample was prepared based on the American Association for Cereal Chemists (AACC)-approved method 10–90 [30] (Table 1). The creaming method was used for batter production in which xylitol, cake gel and sunflower oil were mixed together using a planetary mixer (KSM90, Kitchenaid, Greenville, OH, USA) at high speed until a fluffy and light texture was obtained. Accordingly, water, whole liquid eggs, glycerin, salt, liquid sorbitol, potassium sorbate and flavoring agents were added to the creamed mixture and mixed at middle shear force for 5 min. Finally, flour and baking powder were added to the resulting mixture and mixing continued for 60 s at middle shear force (middle speed) until a viscous and homogeneous batter was resulted [31].

**Figure 1:**
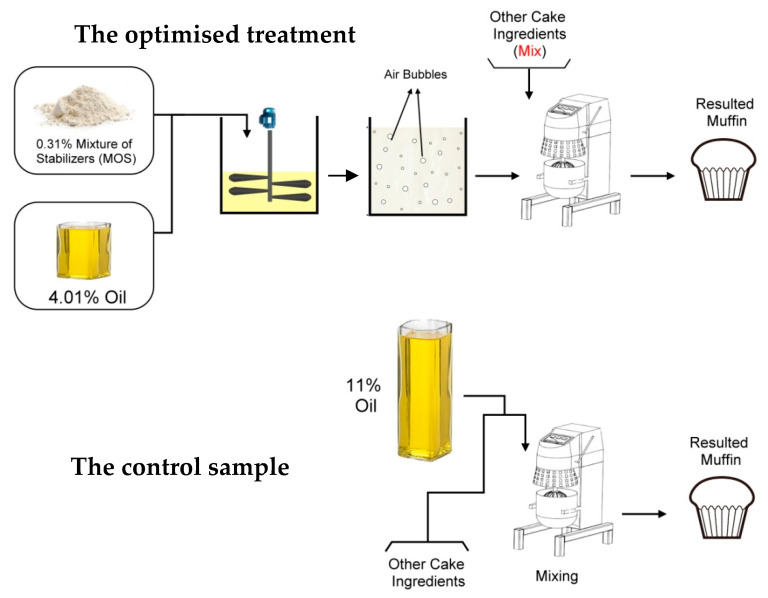
Graphical diagram of the research work.

In order to prepare the treatments, the oil content of the formulation was decreased and replaced by the prepared mixture of stabilizers including protein and hydrocolloids and water according to the experimental plan (Figure 1). The batter was added in a portion of 70 g to each muffin cup and was baked at 180 °C for 35 min in an electric oven (S400, Sveba dahlen, Sweden). The cakes were then cooled down to room temperature and stored at an ambient temperature in a closed container until use. The same procedure was applied to produce other treatments

### 2.4. Experimental Design and Statistical Analysis

The response surface methodology (RSM) is a collection of mathematical and statistical techniques which is used in the development of a functional relationship between a response of interest, Y, and a number of control or input variables, represented by x_1_, x_2_, …, x_k_. 

In this case study, sugar-free, reduced-fat muffin cakes were statistically evaluated using RSM by means of the central composite rotatable design (CCRD). Independent factors included mixture of stabilizers (MOS) soy protein isolate, konjac and guar gums, water and oil. After determination of the range of each independent factor (Table 2), five levels of each independent factor were examined and 13 treatments were designed, including the analysis of variance (ANOVA) and significance mean level (Duncan). The Design-Expert (V. 10) software, Stat-Ease Inc., Minneapolis, MN, USA, was used in this research.

The predicted results were measured using Equation (1) as below:

(1) $$Y=\;\beta_{0}+\beta_{1x1}+\beta_{2x2}+\beta_{12x1x2}+\beta_{11x_{1}^{2}}+\beta_{22x_{2}^{2\;}+\;\varepsilon }$$

where *Y* is the predicted value of the response variables, and *β*_0_, *β*_1_, *β*_2_, *β*_11_, *β*_12_, and *β*_22_ are the constant value, linear coefficient, linear coefficient, quadratic coefficient, interaction coefficient, and quadratic coefficient, respectively. *χ*_1_ and *χ*_2_ are oil and MOS (%) in the batter, respectively, and ε is the random error of the calculation.

### 2.5. Batter Characteristics

#### 2.5.1. Microstructure Analysis of Cakes by Confocal Laser Scanning Microscopy (CLSM)

The CLSM was performed according to the following procedure: to prepare the markers (fluorophores), in order to visualize the fat (with Nile Red, Sigma Aldrich, Poland) and protein (with Alexa Fluor 546, Biocompare), separately. A solution containing 0.5 mg/mL of Nile Red and 0.17 mg/mL of Alexa was prepared in dimethyl formamide as solvent. The fluorophores were kept and protected from light at −20 °C to avoid any possible degradation, and the required amount of solution was prepared on the day of the experiment. These samples (4 g of cake crumb) were placed in containers and frozen the night before the cutting process. Each sample was a 20 μm-thick slice which was cut off in the cryostat microtome chamber (HM 500 OM, Microm International, Walldorf, Germany) set at −25 °C. Before the cover slip was deposited on the top, slices were covered with 15 μL of the fluorophore solution. Mounted slides were stored for 24 h before observation to ensure diffusion of the fluorophores. For excitation purposes, a Nikon (Model A1) CLSM with a green laser at 561 nm was used. The fluorescence emission of the two dyes was detected between 563 and 643 nm and spectral de-convolution was performed. The magnification of images was 20×.

Surface porosities and texture parameters of the cake crumb were analyzed by MATLAB R2018b software (Mathworks Inc., Natick, MA, USA), using the resulted images from CLSM. Eight-bit (gray scale) images were generated from color images using the software and contrasts of images were magnified [32]. Porosity was calculated as the ratio of the porous area to the total area. This method determines the number of gray levels of the image pixels. Five image texture characteristics, including energy, contrast, correlation, homogeneity and entropy, were computed from the gray-level co-occurrence matrices of each sample. The features of the image texture were calculated based on Equations (2)–(6): [28].

(2) $$Energy=\sqrt{\underset{i}{\sum }\underset{j}{\sum }p_{d,\;\theta }{}^{2}\left(i,\;j\right)}$$

(3) $$Contrast=\;\overset{d}{\underset{n=0}{\sum }}n^{2}\left(\underset{i}{\sum }\underset{\left|i-j\right|=n}{\underset{i}{\sum }p_{d,\;\theta }\left(i,j\right)}\right)$$

(4) $$Correlation=\;\frac{\sum_{i}\sum_{j}p_{d,\;\theta }\left(i,j\right)-\;\mu^{2}}{\sigma^{2}}$$

(5) $$Homogenity=\;\underset{i,j}{\sum }\frac{p_{d,\;\theta }\;\left(i,\;j\right)}{1+\left|i-j\right|}$$

(6) $$Entropy=\;-\sum_{i}^{\;}\sum_{j}^{\;}p_{d,\;\theta }\left(i,\;j\right)\log\left(p_{d,\;\theta }\left(i,\;j\right)\right)$$

where $p_{d,\;\theta }\left(i,\;j\right)$ counts the number of neighboring pair pixels with gray values of *i* and *j* at a particular distance of *d* and *a* given direction of *θ*. *µ* and *σ* are mean and standard deviation of $p_{d,\;\theta }\left(i,\;j\right)$, respectively.

#### 2.5.2. Moisture Content

The moisture content of muffins was determined by AACC method 44-15.02 [20,33]. All tests were performed in triplicate for both treatments on day 1 and day 15.

#### 2.5.3. Water Activity

The water activity (a_w_) was determined using a Novasina analyzer (Model Labmaster, Switzerland) at 25° [16]. The measurement was done at day 1 and 15 after baking.

#### 2.5.4. Crumb Texture

Texture profile analysis (TPA) of the crumb of each samples were determined on 1 and 15 days after baking using Brookfield texture analyzer (CT3, Brookfield Engineering Laboratories, Middleboro, MA, USA) with a 4500 g load cell and a 35 mm diameter cylindrical aluminum probe. The samples were cut horizontally in 30 mm-thick × 30 mm-diameter from the central part after the crust had been removed. The tests were performed using the following protocol—two cycle compression tests: 2 mm/s pre-test speed, 2 mm/s test speed, 2 mm/s post-test speed and a penetration distance of 12 mm (40% strain). The quantities of parameters obtained from the force-time diagram (TPA curves) were hardness and cohesiveness and springiness. All tests were performed in triplicate from the same muffins for both treatments on day 1 and day 15.

#### 2.5.5. Specific Volume

Volumes of muffin cakes were evaluated using the seed displacement method according to the AACC standard using millet seeds [33]. The samples were weighted by a digital balance (A&D, model GF-200, Tokyo, Japan) and the specific volume was measured by division of volume to weight of the cakes. All tests were carried out in three replications for both treatments on day 1 and day 15.

#### 2.5.6. Color

The color of muffins crumbs was measured using an Ultra Scan PRO colorimeter (Hunter Associates Laboratory, Inc., Reston, VA, USA). The total amount of color reflected by the sample was defined in accordance with the CIELAB system with D65 illuminator at 10° observer angle. The measured parameters were a^∗^ (greenness (–), redness (+)), and b^∗^ (blueness (–), yellowness (+)), L^∗^ (lightness: 0 = black, 100 = white). Three samples from each batch were measured at one point [34,35]. The total difference in color (ΔE*) among treatments was measured using Equation (7):

(7) $$\Delta E^{*}={\left[{\left(\Delta L^{*}\right)}^{2\;}+{\left(\Delta a^{*}\right)}^{2}+{\left(\Delta b^{*}\right)}^{2\;}\right]}^{\frac{1}{2}}$$

Only ΔE^*^ ≥ 3 means that differences are visible to the human eye.

#### 2.5.7. Sensory Analysis

The acceptance test was used for determining the quality of the sugar-free muffin cake. Sensory evaluations of the muffin cake were conducted using 15-member semi-trained panelists who are familiar with cakes. Panels evaluated the taste, crumb color, hardness, cohesiveness and overall acceptability. For the panelist, there was waterb available to rinse the mouth between tasting the samples [36]. The 9-point hedonic scale was used, where 1 and 9 represented extremely disliked and extremely liked, respectively.

#### 2.5.8. Optimization and Verification

Optimum basic level of ingredients including MOS and oil were evaluated by superimposing the plots for all response variables [37]. The optimum formulations were selected and used for the calculation of predicted responses of variables for optimum treatment. To verify the acquired optimal levels of ingredients by RSM, the optimized treatment was prepared and experimentally analyzed and the results were compared statistically to the predicted values. Comparison between the control and optimized treatment was conducted with three statistical analyses including the analysis of variance (ANVOA) and significance mean level (Duncan) at the significant level of *p* < 0.05 via SAS software version 9.2 (SAS Institute, Cary, NC, USA).

## 3. Results and Discussion

### 3.1. Experimental Design and Model Fitting

The results of ANOVA and lack-of-fit test, regression coefficients with R^2^, adjusted R^2^ and predicted R^2^ are presented in Table 3. R^2^ is the amount of variation around the mean explained by the model, the adjusted R^2^ is the same index adjusted for the number of terms in the model, while predicted R^2^ is a measure of the amount of variation explained by the model in new predicted data.

All *p*-values seemed to be below (*p* < 0.05), which means values were significant and all lack-of-fit amounts were found to be insignificant, which addresses to the correctness of the model. Additionally, almost all R^2^ parameters are more than 0.9, which demonstrates the acceptability of the model. The types of models in the case of being linear, 2Fi or quadratic are shown in Table 3.

### 3.2. Moisture Content

**Figure 2:**
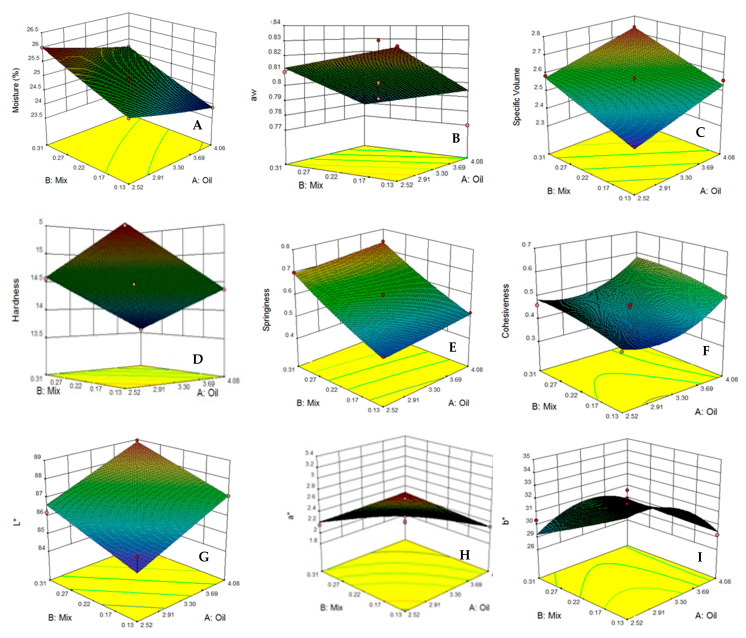
Plots related (**A**) Moisture, (**B**) a_w_ (water activity), (**C**) Specific volume, (**D**) Hardness, (**E**) Springiness, (**F**) Cohesiveness, (**G**) Lightness or L*, (**H**) Redness or a* and (**I**) Yellowness or b* related to the sugar-free muffin cakes as a function of oil and mixture of stabilizers (MOS) content.

The moisture content of cake is defined as the total amount of water that exists in the texture of a cake. As the moisture content of cake increases; it becomes easier and more pleasant to swallow. According to Table 4, the moisture contents of the prepared muffin samples ranged from 23.42 to 25.99 on the first day of storage, and were observed to range from 19.37 to 21.14 on day 15. As the results show, the effect of independent variables (mixture of stabilizers and fat content) on the moisture content of the produced cakes was significant (*p* < 0.05) during storage until day 15. Treatments with higher amounts of stabilizers but the same fat content resulted in a higher moisture content after 15 days of storage. However, the primary moisture contents of treatments were lower, which is due to the barrier effect of the stabilizer mixture against water loss (treatments 7 and 8 in Table 4) [38]. These results are in agreement with those reported by Fernandes and de las Mercedes Salas-Mellado [15], who showed that chia seed mucilage has the ability to absorb water and can replace up to 50% of the fat in high-moisture content bread and cakes. The results were modeled using RSM. Coefficients related to each independent parameter proved the above results. Moreover, the right amounts of R^2^ and lack of fit show the applicability of the chosen model (Table 3). Figure 2A displays the predicted surface plot by RSM. It can be seen that an increase in the percentage of mixture of stabilizers increased the moisture content of cake, which could be related to the high water binding and water holding capacity of hydrocolloids and the protein in the mixture. It is hypothesized that hydrocolloids and the protein, due to having lots of hydroxyl groups, highly tend to bond with water molecules via the formation of hydrogen bonds. Therefore, the viscosity of the batter would be increased and more water molecules would be involved, which resulted in the formation of internal cell walls. As a result, the motion and evaporation of water molecules became difficult, which directly affects the final structure [6].

In the same way, Giacomozzi et al. [34] showed that using monoglycerides oleogels as fat replacers resulted in a higher moisture content. By increasing in the fat content due to the reduction in the water content of the recipe, the moisture content of final treatments decreased. In this respect, the highest and the lowest amounts of moisture were indicated for treatments 5 (with the highest water content) and 4 (with the highest of fat content) on the first day of storage, respectively.

### 3.3. Water Activity

Water activity is defined as water that is not bonded to the substrate, and hence accessible for microorganisms, and therefore is an essential factor that may influence the shelf life of food products. As shown in Table 4, the highest and lowest amounts of water activity on day 1 of storage were seen for treatments 7 (with 0.09% stabilizer and 3.30% fat content) and 4 (with 0.31% stabilizer and 4.08% fat content) and on day 15, were seen for treatments 5 (with 0.22% stabilizer and 2.20% fat content) and 4 (with 0.31% stabilizer and 4.08% fat content), respectively. The most similar treatment to the control sample was treatment 8 (with 0.35% stabilizer and 3.30% fat content) on days 1 and 15, respectively. The predicted models present a suitable prediction possibility, with an R^2^ of 0.882 and 0.844 and a lack of fit of 0.1748 and 0.3748 regarding days 1 and 15, respectively (Table 3). Moreover, according to Figure 2B, similar results for moisture content were observed, indicating that the water activity decreased as the fat content increased (*p* < 0.05). However, increasing the stabilizer content in the mixture had a reverse effect on the water activity (*p* < 0.05). Generally, storage may result in decreasing in the water activity due to water loss and a decrease in moisture content as well as the interaction of water in a retrogradation reaction in the finished product. Due to the high water absorption capacity of the stabilizer mixture, the available water for microorganisms decreases, which results in a decrease in the water activity of samples. Similar results were obtained by Fernandes and de las Mercedes Salas-Mellado [15] and Nourmohammadi and Peighambardoust [27], who reported that the application of chia seed mucilage in reduced-fat cakes resulted in the decrease in free water, and hence the reduction in water activity, increasing the shelf life of the cakes.

### 3.4. Specific Volume

Table 4 shows the obtained results for the cake-specific volume in the reduced-fat samples containing different content ratios of fat and stabilizers. It was indicated that as the fat content decreased, the cake volume reduced; meanwhile, an increase in the content of the mixture of stabilizers resulted in an increase in the cake volume. These results are in agreement with those found by Akesowan [23], which showed that a reduction in fat content will be result in lower volume. The specific volume of the cakes containing hydrocolloid systems depends on the capability of the dough to entrap the air during baking, and also the effect of hydrocolloids on the starch gelatinization temperature. In fact, the effect of hydrocolloids on the volume of cakes is due to an increase in batter viscosity, which helps to keep the gas inside the cake during the initial stage of baking process. Due to the competition between hydrocolloids and starch for the absorption of free water, the gelatinization temperature of starch increases. This condition leads to a latency in the transformation of aerated-liquid batter into a solid-porous structure. Consequently, there will be more time for the cake to expanded [39,40]. As is apparent in Figure 2C, the specific volume increased as a result of an increase in the amount of MOS and decreased with the reduction in oil content. Similar results were also obtained by Román et al. [22], which indicated that by using extruded wheat flour as a fat replacer, the final volume of cake increased as a result of the increasing stability of the bubbles in the batter. Hence, the highest and lowest volumes regarding the treatments were seen in treatments 4 and 1, respectively. As is apparent in Table 3, the models for the prediction of cake volume on days 1 and 15 of storage showed a high R^2^ and an insignificant lack of fit (*p* > 0.05). Accordingly, these parameters showed the accuracy of the model.

### 3.5. Hardness

The hardness of a cake is defined as the maximum applied force at the first bite. The hardness of a cake depends on moisture, a_w_ and the fat content, and all these factors are affected by the cake formulation and recipe. Two ingredients of cake recipes include fats and stabilizers, and the amounts of stabilizers are completely dependent on the water content. The statistical results show that the hardness of the cakes produced by the treatments decreased significantly (*p* ≤ 0.01) by increasing the amount of oil and MOS (Table 4 and Figure 2D), which was in accordance with the findings by Gharaie et al. [41], which showed that the existence of hydroxyl groups in the structure of hydrocolloids and their reaction with water resulted in an increase in water binding capacity via the creation of hydrogen bonds, which will be result in the reduction in the hardness of the final product. Fats and oils, due to their plasticizing role and by penetrating into the protein and carbohydrate chains, cause the easing of the motion of these chains in relation to each other, and as a result, the amount of textural hardness decreases [42]. Although the change in hardness on day 15 was the same as that on day 1, due to the passage of time from day 1 to day 15, the hardness of the texture increased due to the decrease in water content. As shown in the models according to Table 3, the existence of minus coefficients of models demonstrates the reverse effects of the evaluated parameters on the hardness of the texture. Additionally, the increase in the coefficients on day 15, in contrast to day 1, represents the increase in hardness due to the passage of time during the shelf life. R^2^ was 0.996 and 0.983 and lack of fit was 0.3258 and 0.7707 for days 1 and 15, respectively, showing the suitability of the suggested models (Table 3). The trend of the change in the hardness of the cake produced by different treatments is presented in Figure 2D, alongside the changes in fat and MOS contents.

### 3.6. Springiness

The springiness of the cake refers to the rate of deformation recovery at which a deformed piece of cake goes back to its undeformed condition just after the applied deforming tension has been removed [7]. According to the results of springiness tests which are shown in Table 4 and Figure 2E, it was shown that an increase in oil and MOS content resulted in a significant increase in the springiness on days 1 and 15 (*p* < 0.05), which could be assumed to be a result of the increase in the number of air bubbles and the cake matrix expansion [43]. The effect of MOS on increasing the springiness was greater than the oil, since the mixture of proteins and hydrocolloids created a network which helped to retain the air bubbles in the cake matrix. Moreover, this could be due to the capability of konjac and guar gums to imprison a large amount of water [44]. The highest amount of springiness on day 1 could be seen in treatment 8 with 0.35% stabilizer and 3.30% oil (Table 4). On day 15, similarly to day 1, with the increase in MOS and fat content, springiness increased. However, the results show that with the passage of time from day 1 to day 15, the amount of springiness decreased, which could be due to the slight water loss during the storage time. The relevant models for days 1 and 15 have a R^2^ value of 0.995 and a lack of fit value of 0.0324 for day 1 and R^2^ and lack of fit values of 0.987 and lack of fit 0.8544 for day 15, respectively, which are presented in Table 3. 

### 3.7. Cohesiveness

Cohesiveness refers to the internal bond strength of a food structure that is dependent on the structure’s ability to withstand deformation [36]. The cohesiveness was seen to be variable from 0.311 to 0.594 in day 1. Treatments 1 and 7 produced the most cohesiveness and treatment 4 produced the least cohesiveness. The trend of changes in cohesiveness on day 15 was similar to that on day 1 (Table 4). It was seen that cohesiveness has an indirect correlation with both oil and MOS content (*p* < 0.05), meaning that as the contents of these two ingredients increased, the number of air cells, followed by the texture porosity, rose, which resulted in less cohesiveness and lower required energy for the compression of the cakes produced by the treatments (Table 4 and Figure 2F) [36]. This finding could be due to the formation of voids in the texture, and these hollow spaces resulted in a disjointed structure, and consequently, less cohesiveness was acquired [45]. Table 3 shows the presented model for cohesiveness, with R^2^ and lack of fit values of 0.941 and 0.2566 for day 1 and 0.920 and 0.1366 for day 15, respectively. The higher coefficient for MOS as a fat replacer shows that the impacts of fat replacers are higher than oil regarding a decrease in cohesiveness.

### 3.8. Color

The lightness value represents the amount of brightness and refers to the darkest black at L* = 0, and the brightest white at L* = 100. According to Table 4 and Figure 2G, it is clear that by increasing the content of the mixture of stabilizers, the L* value increased, whilst this parameter decreased with increasing the amount of oil. It is hypothesized that hydrocolloids have the ability to aerate and entrap air, which results in the whitening of the batter and, subsequently, a brighter texture of the final product [14,46,47]. Therefore, highest values of L* were seen for treatments 4 (MOS: 0.31 and oil: 4.08) and 6 (MOS: 0.22 and oil: 4.40), and the lowest values were seen in treatments 1 (MOS: 0.13 and oil: 2.52) and 5 (MOS: 0.22 and oil: 2.20) based on Table 4. The same trend was seen regarding treatments on day 15 of storage but to a lower degree, which is assumed to be due to the water loss of treatments during storage, since water plays a great role in the brightness of food substances [48]. The results of statistical analysis on the lightness value produced by treatments are presented in Table 3, which shows that the models for the prediction of cake brightness on days 1 and 15 of storage show a high R^2^ (0.998 and 0.995 for days 1 and 15, respectively) and an insignificant lack of fit (0.0715 and 0.6079, respectively). Statistical analysis shows that changing the MOS and oil content in the cake recipe has a significant linear correlation with the lightness value (Table 3).

a* and b* refer to the amount of redness and blueness colors existing in the substrate, respectively. According to Table 4 and Figure 2H,I, it is clear that increasing the oil content resulted in a reduction in both a* and b*, as the mixture of gums, oil and water results in oleogels, which are whitish due to their high capacity for aeration during their production using a high shear force [46]. On the other hand, increasing the amount of MOS had a negative effect on these two parameters [49]. Therefore, the highest values of a* and b* among the treatments was seen for treatment 5, and the lowest values of these two parameters were seen for treatment 2 (Table 4). On day 15 of storage, from the quantitative point of view, higher results were obtained, which is hypothesized to be due to the water loss of treatments during storage. The R^2^ of a* and b* for days 1 and 15 were seen to be 0.990 and 0.989 and 0.945 and 0.989, and regarding lack-of-fit, the values were seen to be 0.0780 and 0.0580 and 0.2325 and 0.0523, respectively (Table 3).

ΔE* is the parameter combining all color-related values—L*, a* and b*—according to Eq. 1 to evaluate the total color change. The greatest and the least amount of color change were seen for treatments 6 (MOS: 0.0.22 and oil: 4.40) and 5 (MOS: 0.22 and oil: 2.20), respectively. The roles of MOS and oil in color change are opposite to each other. Hence, the equilibrium between the two values determines the amount of ΔE*.

### 3.9. Optimization

The main purpose of the response surface methodology is to reach the best conditions for a process to obtain the optimum responses [31]. The optimization of physico-chemical parameters, including moisture content, water activity, L*, a*, b*, hardness, springiness, and cohesiveness was performed based on the desired quantities (Table 5). The determinations showed that the optimum percentage of MOS to replace oil for both day 1 and day 15 was 0.31 and 4.08, respectively, which shows 62.9% reduction in consumed oil. The new treatment was prepared and compared to the prediction models, which revealed that there were no significant differences (*p* < 0.05) among the predicted quantities and the obtained results (Table 5), which indicates the correctness of the chosen model.

### 3.10. Microstructural Characteristics of Cake Crumb

**Figure 3:**
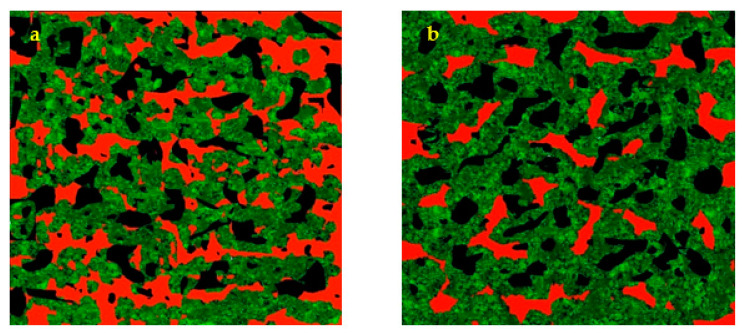
CLSM- Confocal Laser Scanning Microscope images of control (**a**) and optimized (**b**) muffins.

The resulting CLSM (Confocal Laser Scanning Microscope) images of the control and optimized muffin cakes are shown in Figure 3. By evaluating the images, it was obvious that the control cake (a) contains higher amounts of oil, while the red areas in the optimized cake (b) were smaller than in the control cake. Moreover, in the optimized cake, green areas (proteins) were more common than in the control sample, which could be due to using the soy protein isolate. 

To evaluate the capability of MOS in entrapping the air bubbles (black areas) in comparison to the oil, image processing was applied. The results of image processing are shown in Table 6, showing no significant difference in the case of any of the parameters, including porosity, energy, contrast, homogeneity and entropy. Energy measures the number of repeated pixel pairs, contrast measures the local gray variation in an image, homogeneity reveals the uniformity of texture within an image, and entropy defines the randomness within the image.

### 3.11. Sensory Evaluation

**Figure 4:**
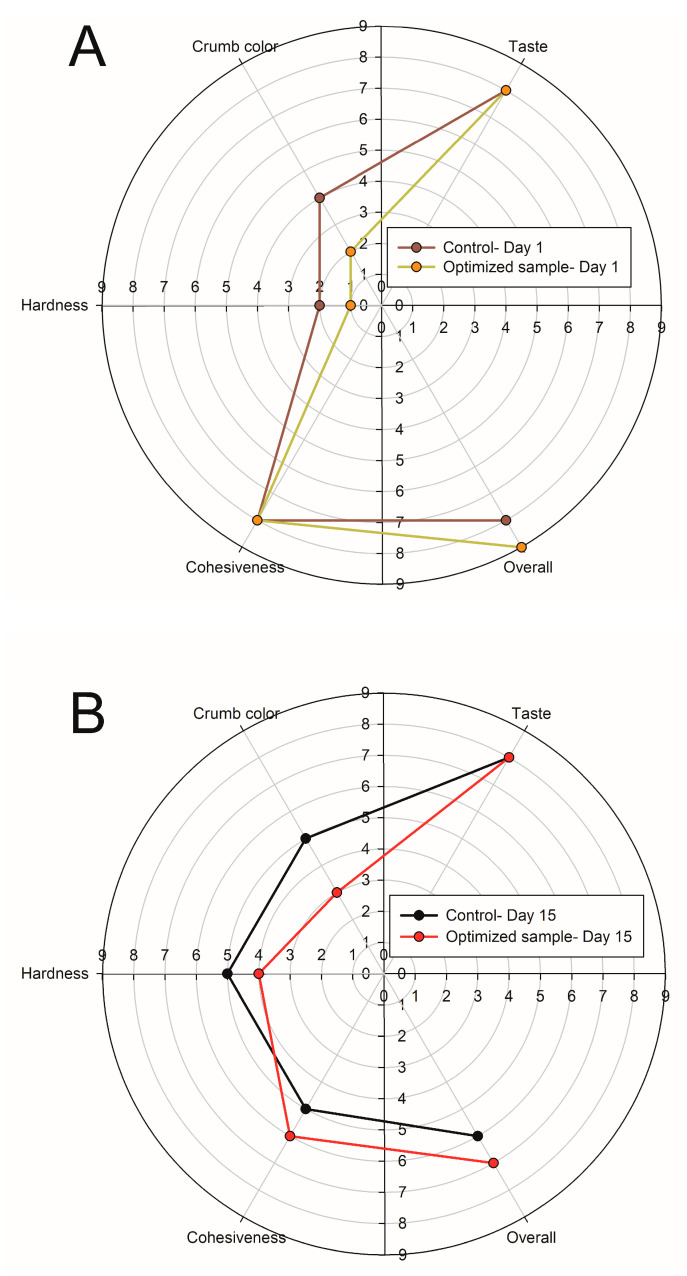
Organoleptic characteristics of the control and optimized muffins on day 1 (**A**) and day 15 (**B**) after their production.

The average results of the sensory evaluation for sugar-free muffin samples after 1 and 15 days of storage are summarized in Figure 4A,B. The optimized muffin had the lowest hardness and crumb color after 1 day of storage. Additionally, this sample showed the highest level of taste, cohesiveness, and overall acceptance (Figure 4A). Regarding the taste and cohesiveness parameters, the optimized muffin acquired the same score as the control. The hardness was increased after 15 days of storage, though this value on day 15 of storage was low in the optimized muffin in comparison with the control sample (Figure 4B). As indicated in Figure 4B, the panelists assigned the highest score for cohesiveness and overall acceptability to the optimized muffin. The crumb color of the optimized muffin on day 15 of storage was also lighter than the color of the control muffin. The taste value had a similar score in both muffins (Figure 4B).

## 4. Conclusions

Considering the popularity of bakery products, especially cakes, the consumption of these products is growing day by day. However, on the other hand, they contain a high number of calories, which could result in obesity. Therefore, the culture of eating habits is shifting towards healthier products which deliver less sugar and fat to the consumer. Hence, more people tend to buy products which contain lower amounts of sugar and fat, while maintaining acceptable textural and sensorial features. In this view, this research focused on the possible replacers for the oil content of muffin cakes. To achieve this purpose, a mixture of stabilizers in combination with isolate soy protein was used to decrease the oil usage in the cake recipe. Based on the findings of this study, increasing the MOS content resulted in an increase in moisture content, water activity, specific volume, height, springiness, cohesiveness, chewiness and L* values, but had a negative effect on hardness, a* and b* values.

By optimizing the usage amount of oil and MOS in the recipe for muffins, it was concluded that a 62.9% reduction in oil content without any significant differences (*p* < 0.05) in the final product was obtained. The optimized muffin demonstrated the lowest value in hardness and crumb color and the highest value in overall acceptance, respectively.

## Figures and Tables

**Table 1:** Recipe of the control muffin cake.

%	Ingredients
29.47	Flour
17.5	Water
17	Xylitol
15	Whole pasteurized egg
11	Sunflower oil
5	Liquid sorbitol
2	Glycerin
1.3	Cake gel
1.2	Baking powder
0.03	Potassium sorbate
0	Powder emulsion mixture
0	Emulsion water
0.3	Salt
0.2	Flavorings

**Table 2:** Coded symbols and levels of independent variables.

Independent Variable	Coded Symbols	Levels				
		−1.41	−1	0	+1	+1.41
Oil	*χ*_1_	2.20	2.52	3.30	4.08	4.40
MOS	*χ*_2_	0.09	0.13	0.22	0.22	0.35

**Table 3:** ANOVA and model fitting for the response variables of the central composite design.

	Moisture (%)		Water Activity		Specific Volume (cm^3^/g)		Hardness (N)		Springiness		Cohesiveness		L*		a*		b*	
Day																		
	1	15	1	15	1	15	1	15	1	15	1	15	1	15	1	15	1	15
**Sequential model sum of squares (*p*-values)**																		
Linear	<0.0001	<0.0001	0.003	<0.0001	<0.0001	<0.0001	<0.0001	<0.0001	<0.0001	<0.0001	0.0664	0.059	<0.0001	<0.0001	<0.0001	<0.0001	<0.0001	<0.0001
2FI	0.9024	0.8196	0.9041	0.566	0.425	0.5233	1	0.0391	1	0.8021	1	0.9651	0.8946	0.7143	0.5216	0.173	0.51	0.5257
Quadratic	0.0034	<0.0001	0.9634	0.8418	0.6591	0.5477	0.6019	0.2283	**0.0262**	0.2423	0.0003	0.0026	0.006	0.011	0.0246	0.4296	0.0253	0.0281
**Lack-of-fit Tests (*p*-values) of significant model**																		
Linear	0.0414	0.0014	0.1748	0.3748	0.1659	0.3258	0.7707	0.0324	0.1759	0.8544	0.0094	0.0151	0.01	0.15	0.0268	0.2325	0.0201	0.0194
2FI	0.0312	0.001	0.1368	0.3253	0.1503	0.2845	0.7006	0.0624	0.1374	0.7923	0.0069	0.0112	0.0074	0.1197	0.0219	0.2598	0.0165	0.0158
Quadratic	0.3159	0.4086	0.0688	0.1944	0.0941	0.2095	0.5946	0.0663	0.4589	0.9751	0.2566	0.1366	0.0715	0.6079	0.078	0.2146	0.058	0.0523
X_1_	<0.0001	0.0002	0.0181	0.0027	<0.0001	<0.0001	<0.0001	<0.0001	0.0002	0.0005	0.0006	0.0007	<0.0001	<0.0001	<0.0001	<0.0001	<0.0001	<0.0001
X_2_	<0.0001	<0.0001	0.0047	0.0026	<0.0001	<0.0001	<0.0001	<0.0001	<0.0001	<0.0001	0.0057	0.0057	<0.0001	<0.0001	<0.0001	0.0003	<0.0001	<0.0001
X_1_X_2_	0.8438	0.2677	0.9156	0.606	0.4598	0.5434	1	0.034	1	0.784	1	0.9286	0.8001	0.5456	0.3513	0.1985	0.3404	0.3644
X_1_^2^	0.0043	<0.0001	0.9833	0.6166	0.5241	0.378	0.5088	0.3675	0.6548	0.7908	0.0002	0.001	0.0036	0.0127	0.0244	0.2277	0.0248	0.0266
X_2_^2^	0.006	0.0116	0.7965	0.7193	0.5883	0.61	0.4163	0.1202	0.009	0.1038	0.109	0.0777	0.0116	0.0167	0.0357	0.5845	0.0371	0.0414
**Predictive parameter of significant model**																		
R2	0.999	0.997	0.882	0.844	0.984	0.983	0.996	0.983	0.995	0.987	0.941	0.92	0.998	0.995	0.99	0.945	0.989	0.989
Adjusted R2	0.998	0.994	0.757	0.812	0.979	0.977	0.995	0.977	0.991	0.985	0.899	0.863	0.996	0.991	0.982	0.934	0.981	0.981
Predicted R2	0.995	0.985	0.596	0.71	0.966	0.967	0.994	0.941	0.98	0.982	0.713	0.559	0.987	0.982	0.939	0.898	0.934	0.931

**Table 4:** Experimental design and physicochemical properties of muffin cake treatments on days 1 and 15.

Treatments	MOS %: Oil %: Water %	Crumb Moisture (%)		Crumb Water Activity		Specific Volume (cm^3^/g)		Hardness (N)		Springiness		Cohesiveness		L* crumb		a* crumb		b* crumb		ΔE* crumb	
		Day																			
		1	15	1	15	1	15	1	15	1	15	1	15	1	15	1	15	1	15	1	15
Blank	0:11:00	25.71	23.33	0.811	0.78	2.66	2.47	13.59	17.28	0.711	0.487	0.315	0.165	83.27	80.65	5.32	5.51	40.91	42.01	0	0
1	0.13:2.52:8.35	25.79	20.27	0.835	0.821	2.39	2.31	15.34	19.44	0.482	0.339	0.584	0.437	84.65	82.75	4.11	4.23	31.59	32.24	9.5	10.07
2	0.13:4.08:6.79	23.88	19.37	0.788	0.77	2.56	2.48	14.36	17.95	0.522	0.391	0.456	0.302	88.19	86.94	2.15	2.19	20.72	21.12	21.02	22.07
3	0.31:2.52:8.17	25.22	21.98	0.832	0.811	2.59	2.51	14.55	18.35	0.703	0.555	0.414	0.271	85.32	83.57	4.19	4.3	32.24	32.78	8.98	9.76
4	0.31:4.08:6.61	23.71	20.5	0.746	0.728	2.73	2.65	13.57	17.29	0.746	0.601	0.311	0.169	89.04	88.12	2.34	2.37	23.99	24.29	18.12	19.48
5	0.22:2.20:8.58	25.99	21.14	0.847	0.828	2.44	2.37	15.22	19.13	0.563	0.432	0.508	0.349	85.16	83.29	4.31	4.42	33.16	33.7	8.04	8.79
6	0.22:4.40:6.38	23.42	20.03	0.765	0.749	2.66	2.58	13.75	17.61	0.621	0.495	0.405	0.252	88.55	87.23	2.22	2.25	23.17	23.48	18.77	19.93
7	0.09:3.30:7.61	25.04	19.65	0.849	0.825	2.39	2.31	15.06	18.89	0.456	0.321	0.594	0.451	85.62	83.02	3.98	4.04	30.61	30.8	10.65	11.55
8	0.35:3.30:7.35	24.67	21.5	0.801	0.785	2.68	2.6	13.9	17.75	0.77	0.632	0.352	0.211	87.91	86.54	3.84	4.01	29.53	30.57	12.38	12.95
9	0.22:3.30:7.48	24.91	20.53	0.827	0.811	2.57	2.49	14.46	18.35	0.594	0.463	0.459	0.316	86.54	86.55	3.92	3.99	30.17	30.51	11.31	13.01
10	0.22:3.30:7.48	24.88	20.49	0.828	0.812	2.55	2.48	14.45	18.33	0.603	0.473	0.463	0.32	86.51	86.65	3.95	4.02	30.39	30.69	11.09	12.9
11	0.22:3.30:7.48	24.87	20.48	0.827	0.81	2.55	2.47	14.52	18.45	0.585	0.459	0.458	0.305	86.54	86.75	3.97	4.03	30.51	30.72	10.99	12.92
12	0.22:3.30:7.48	24.88	20.49	0.828	0.811	2.54	2.46	14.48	18.39	0.598	0.462	0.454	0.312	86.53	86.46	3.96	4.03	30.46	30.74	11.03	12.77
13	0.22:3.30:7.48	24.84	20.41	0.825	0.81	2.54	2.47	14.55	18.41	0.608	0.473	0.457	0.319	86.44	86.55	3.89	4.06	30.04	30.79	11.41	12.76

**Table 5:** Optimized quantities of physicochemical parameters of muffin cakes.

Parameter	Aim	Day-1					Day-15				
		Lowest Quantity	Highest Quantity	Predicted Quantity	Obtained Quantity	Control Sample	Lowest Quantity	Highest Quantity	Predicted Quantity	Obtained Quantity	Control Sample
Moisture (%)	In range	23.42	25.99	25.22	25.92	25.71	19.37	21.50	21.38	22.21	22.33
aw	Min.	0.746	0.849	0.82	0.83	0.81	0.791	0.809	0.79	0.81	0.78
Specific volume	Max.	1.90	2.15	2.06	2.11	2.21	1.85	2.20	2.01	2.09	2.17
L*	In range	83.45	90.52	87.88	88.53	88.10	81.75	88.79	86.07	87.14	87.43
a*	In range	2.15	3.41	3.03	3.06	3.08	2.25	3.48	3.15	3.19	3.13
b*	In range	25.73	40.91	36.34	35.29	34.98	25.77	40.95	36.37	36.97	36.34
Hardness	Min.	13.57	15.34	13.57	13.21	13.59	17.29	19.44	17.40	17.95	17.28
Springiness	In range	0.45	0.77	0.72	0.71	0.70	0.321	0.632	0.60	0.63	0.478
Cohesiveness	In range	0.38	0.66	0.57	0.55	0.54	0.218	0.432	0.39	0.37	0.35

**Table 6:** Microstructural features of control and optimized muffins crumbs.

Image Processing Parameters	Muffin Cakes	
	Control	Optimized
Porosity	0.524 ^a^	0.539 ^a^
Energy	0.00048 ± 0.00003 ^a^	0.00044 ± 0.00004 ^a^
Contrast	1729 ± 62 ^a^	1699 ± 56 ^a^
Homogeneity	0.242 ± 0.01 ^a^	0.132 ± 0.012 ^a^
Entropy	12.451 ± 0.7 ^a^	14.842 ± 0.4 ^a^

^a^ Superscripts with same letters indicate not significant difference *p* ≤ 0.05 among treatments. Values are mean ± standard deviation.

## Data Availability

The data presented in this study are available on request from the corresponding author. The data are not publicly available due to privacy.
